# Identification of a novel perifornical-hypothalamic-area-projecting serotonergic system that inhibits innate panic and conditioned fear responses

**DOI:** 10.1038/s41398-024-02769-3

**Published:** 2024-01-25

**Authors:** Cristian S. Bernabe, Izabela F. Caliman, Aline R. R. de Abreu, Andrei I. Molosh, William A. Truitt, Anantha Shekhar, Philip L. Johnson

**Affiliations:** 1 Department of Anatomy, Cellular Biology & Physiology, Indiana University School of Medicine, Indianapolis, IN USA; 2 Stark Neurosciences Research Institute, Indiana University School of Medicine, Indianapolis, IN USA; 3 Department of Medicine, Division of Clinical Pharmacology, Indiana University School of Medicine, Indianapolis, IN USA; 4 Department of Psychiatry, Indiana University School of Medicine, Indianapolis, IN USA; 5 Departamento de Alimentos, Escola de Nutrição da Universidade Federal de Ouro Preto, Ouro Preto, MG Brazil; 6 School of Medicine, University of Pittsburgh, Pittsburgh, PA USA; 7 Department of Biology, University of South Dakota, Vermillion, SD USA

**Keywords:** Neuroscience, Psychiatric disorders

## Abstract

The serotonin (5-HT) system is heavily implicated in the regulation of anxiety and trauma-related disorders such as panic disorder and post-traumatic stress disorder, respectively. However, the neural mechanisms of how serotonergic neurotransmission regulates innate panic and fear brain networks are poorly understood. Our earlier studies have identified that orexin (OX)/glutamate neurons within the perifornical hypothalamic area (PFA) play a critical role in adaptive and pathological panic and fear. While site-specific and electrophysiological studies have shown that intracranial injection and bath application of 5-HT inhibits PFA neurons via 5-HT_1a_ receptors, they largely ignore circuit-specific neurotransmission and its physiological properties that occur in vivo. Here, we investigate the role of raphe nuclei 5-HT inputs into the PFA in panic and fear behaviors. We initially confirmed that photostimulation of glutamatergic neurons in the PFA of rats produces robust cardioexcitation and flight/aversive behaviors resembling panic-like responses. Using the retrograde tracer cholera toxin B, we determined that the PFA receives discrete innervation of serotonergic neurons clustered in the lateral wings of the dorsal (lwDRN) and in the median (MRN) raphe nuclei. Selective lesions of these serotonergic projections with saporin toxin resulted in similar panic-like responses during the suffocation-related CO_2_ challenge and increased freezing to fear-conditioning paradigm. Conversely, selective stimulation of serotonergic fibers in the PFA attenuated both flight/escape behaviors and cardioexcitation responses elicited by the CO_2_ challenge and induced conditioned place preference. The data here support the hypothesis that PFA projecting 5-HT neurons in the lwDRN/MRN represents a panic/fear-off circuit and may also play a role in reward behavior.

## Introduction

Panic is an innate adaptive response to an imminent threat that includes an integrated pattern of behaviors (e.g., fighting or fleeing) accompanied by cardiovascular, respiratory, and endocrine perturbations. One of the pivotal brain areas underlying such responses is the hypothalamic area surrounding the fornix, where electrical stimulation in cats produced panic-associated defense reactions (e.g., piloerection, hissing, and arching of back) as demonstrated in the seminal work of Hess and Brugger [1]. Subsequent studies in rodents determined that pharmacological stimulation or disinhibition of similar anatomical regions, including the perifornical hypothalamus and perifornical part of the lateral hypothalamus (henceforth defined as perifornical hypothalamic area, PFA) elicit behavioral (running/escape) and physiological (blood pressure, tachycardia, thermal changes, and hyperventilation) components of the “fight or flight” response [2–9]. Similarly, stimulation of the PFA during neurosurgeries in humans produced tachycardia, increased blood pressure, thermal sensations, hyperventilation, and self-reports of fear of dying or panic attack (PA) [10, 11].

One potential candidate within the PFA and parts of the dorsomedial and lateral hypothalamus that is involved in the generation of panic responses is the orexin (OX) system [12, 13]. These OX-producing neurons play a critical role in the expression of anxiety- and panic-like responses in rats as panic-associated behaviors and cardiovascular responses elicited by 20% CO_2_ inhalation (a suffocation-inducing stimulus) were attenuated upon pharmacological inhibition of the OX1, but not OX2 receptors [14–18]. More importantly, OX levels were elevated in the cerebrospinal fluid of human subjects with panic compared to controls [16], a selective orexin-1 receptor antagonist reduced CO_2_-induced fear and anxiety symptoms [18], and the dual OX receptor antagonist suvorexant improved anxiety symptoms in insomnia patients in a recent clinical trial [19].

Serotonin (5-HT) plays a critical role in anxiety and psychological trauma-related disorders, such as panic disorder (PD) and post-traumatic stress disorder (PTSD), respectively [(for review, see [20, 21]]. Within the PFA, 5-HT inhibits panic-like responses [22] elicited by local electrical stimulation [23, 24] and GABAergic disinhibition [25], and 5-HT also suppresses stress-induced endocrine responses [26]. OX neurons in the PFA express 5-HT_1A_ receptors [27, 28] and are inhibited by bath application of 5-HT, which can be blocked with a 5-HT_1A_ receptor antagonist [28]. Serotonergic innervation to the PFA mainly originates from the dorsal (DR) and median (MR) raphe nuclei [29, 30]. However, a distinct cluster of serotonergic neurons located within the lateral wings of the DR (lwDR) is hypothesized to functionally reduce panic responses [31–33].

Even though evidence suggests that 5-HT inhibits panic-associated behaviors via the 5-HT_1A_ receptors in the PFA [23, 24], the effects of 5-HT on other physiological aspects of an integrative panic response, such as cardioexcitation, have been largely unexplored, especially in serotonin circuit-specific neurotransmission and its physiological properties that occur in vivo. We hypothesize that optogenetic excitation of serotonergic fibers in the PFA originating from the lwDR will attenuate the physiological and behavioral components of panic-like responses induced by panicogenic/suffocation-related stimuli (7.5% and 20% CO_2_ exposure). Conversely, we also hypothesized that selective lesioning of 5-HT fibers in the PFA will lead to exacerbated physiological and behavioral components of panic-like responses induced by panicogenic/suffocation stimulus (7.5% and 20% CO_2_ exposure). Finally, since the OX system in the PFA projects to the amygdala [12] where it enhances fear-conditioned behaviors [34–36], we predict that selective lesions to the PFA-projecting serotonergic network will facilitate fear-conditioned behaviors.

To test these hypotheses, we used pathway-specific loss- and gain-of-function studies to investigate the role of 5-HT neurons originating from the DR/MR in the context of innate panic-associated behavior, physiological responses, and fear-conditioned behaviors. We utilized wireless optogenetics to excite OX/glutamatergic neurons in the PFA to further confirm its role as a panic-generating site. We then used a gain-of-function approach with wireless optogenetic stimulation to selectively activate the PFA-projecting serotonergic systems while assessing innate anxiety- and panic-associated physiology and behaviors. Finally, in our loss-of-function study, we first injected a selective anti-5-HT transporter-saporin (SERT-SAP) toxin into the PFA and assessed anxiety-, panic-like, and conditioned fear responses, then injected a retrograde tracer into PFA to confirm selective lesioning of the serotonergic projecting raphe nuclei neurons.

## Methods

### Animals and housing conditions and surgical procedures

All experiments were carried out in post-weaning (45–55 g) or adult (300–350 g) male Sprague Dawley rats acquired from Envigo Laboratories (Indianapolis, IN) and housed under standard conditions (12/12 light/dark cycle; lights on at 7:00 AM; 22 °C). Food and water were provided *ad libitum*. Caretaking and experimental procedures were in accordance with the NIH Guide for the Care and Use of Laboratory Animals, Eighth Edition [37], and approved by our Institutional Animal Care and Use Committee. Rats were anesthetized under 2–3% isoflurane delivered through a nose cone, and all stereotaxic coordinates used below were according to a standard stereotaxic atlas of the adult rat brain [38]. All groups were tested in a counterbalanced fashion between 8:00 AM and 12:00 PM.

### Experiment 1: Role of the PFA as a panic-generating site

Post-weaning juvenile male Sprague Dawley rats received local (coordinates in mm, AP: −1.90; ML: ±1.10; DV: −7.20) bilateral 300 nl injections of virus (AAV-CaMKIIa-ChR2-eYFP) expressing enhanced yellow fluorescent protein (eYFP) fused to channelrhodopsin-2 (ChR2) or its control virus (AAV-CaMKIIa- eYFP, University of North Carolina Vector Core, Chapel Hill, NC) that mediates expression of eYFP alone. Viruses were injected with the aid of a glass pipette connected to a pico-injector (PLI-100, Harvard Apparatus, Holliston, MA) set at 100 nl/min. Four weeks after, animals were implanted with wireless bilateral optical fibers (TeleLC-B-8.9-500-2.5, TeleOpto, Tokyo, Japan) aimed at the PFA (AP: −3.00; ML: ±1.25; DV: −7.70). The following week, rats were cannulated in the femoral artery with DSI radiotelemetry probes (HD-S11; New Brighton, MN) for physiological assessments.

One week after DSI probe implantation, rats were habituated to Plexiglas boxes (50 cm width × 30 cm length × 40 cm height) and received photostimulation (470 nm, 20 Hz, 1 mW, 5-ms pulses, 5 min) while general locomotor activity, behavioral, and cardiovascular responses were assessed. The following behaviors were assessed: exophthalmos (characterized by the complete opening of the eyelids, leading to the eyeball protrusion assuming a spherical aspect), immobility (i.e., absence of body movement except breathing), and running [i.e., fast locomotion involving either contralateral swing of fore and hindlimbs (trotting) or the alternation between them (galloping)].

One day after being photostimulated, rats were habituated to an open field apparatus and the next day tested in the social interaction (SI) test, a fully validated test of experimental anxiety-like behavior as described by others [39, 40]. For the SI test, both experimental and an unfamiliar “partner” rats were simultaneously placed into the opposite corners of the open field for the 5 min test. During the SI test, the “experimental” rat received optogenetic stimulation (470 nm, 20 Hz, 1 mW, 5-ms pulses, 5 min). The total duration (in seconds) of non-aggressive physical contact (grooming, sniffing, crawling over and under) initiated by the ‘experimental’ rat was manually scored by an experimenter who was unaware of the experimental groups.

Lastly, we used the real-time place preference/avoidance (RTPP/A) test. The test was performed in four different sessions (20 min each) over three days. During the habituation session (day 1), rats were placed pseudo-randomly on one side of the chamber (starting side was counterbalanced across animals). On day 2 (stimulation session), rats were wirelessly stimulated (blue light) as soon as at least 50% of their body entered the opposite chamber of the apparatus. Stimulation continued until the animal returned to the non-stimulated control side. Rats were exposed to the apparatus without LED stimulation for 45 min and 24 h after the stimulation session. We utilized the difference score (percentage time spent on the stimulation side minus percentage time spent on the non-stimulation side) to determine place preference/avoidance. Rats received a five-minute photostimulation session 90 min prior to euthanasia.

### Experiment 2: Cholera toxin B microinjection

Rats received 100 nl of the retrograde tracer Cholera Toxin B subunit (CTB; 1% w/v in ACSF, List Biological Laboratories, Campbell, CA) over 5 min into the PFA three weeks prior to euthanasia as previously described [41].

### Experiment 3: Transient gain-of-function: effects of optogenetic stimulation of PFA-projecting DR/MR neurons on anxiety- and panic-associated behaviors

We utilized the intersectional genetics approach to selectively target and activate the PFA-projecting DR/MR neurons. Post-weaning juvenile male, Sprague Dawley rats, were first bilaterally injected into the PFA with 300 nl of a retrogradely trafficked canine adenovirus (CAV-2) that expresses Cre-recombinase (CAV-CMV-Cre, Institut de Génétique Moléculaire de Montpellier, France) [42]. In the same surgery, rats also received a 300 nl injection of either a Cre-dependent virus expressing ChR2 (AAV-EF1a-DIO-hChR2-eYFP) or its control (AAV-EF1a-DIO-eYFP, University of North Carolina Vector Core, Chapel Hill, NC) into the DR (AP: −6.60; ML: −1.40; DV: −5.50, 15° oblique to the midsagittal plane), same approach as used by us [41] and others [43, 44]. Virus titers were between 4.3 and 5.1 × 10^12^ pp/ml for all constructs. Four weeks later, adult rats (300–350 g) received bilateral wireless LED optical fiber implants as described above (TeleLC-B-8.9-500-2.5, TeleOpto) aimed at the PFA. The stimulation parameters (470 nm, 20 Hz, 10 mW, 5-ms pulses, 5 min) were chosen based on in vitro and in vivo DR neuronal firing patterns and serotonin release in rodents [45, 46]. One week later, rats were implanted with DSI probes as described above. After recovery, rats received photostimulation in a Plexiglas chamber to assess behavioral and physiological responses as described in “Experiment 1”.

Two days later, rats were allowed to freely explore the elevated plus-maze (EPM) for 10 min. In the first 5 min, photostimulation was paired with the exploratory behavior in the open arm, and for the remaining 5 min rats were allowed to explore the apparatus without photostimulation. Two days later, rats were submitted to four different SI sessions, 48 h apart (i.e., baseline, optical stimulation, bright light, and 20% CO_2_ challenges). Rats received optogenetic stimulation during each SI session (5 min duration), except baseline. The bright light challenge consisted of an abrupt transition from dim red light (40-watt red light, 1 lux) to bright white fluorescent lighting (488 lux) during the SI session [47], whereas the 20% CO_2_ challenge session consisted of pre-exposure to the “experimental” rat to a Plexiglas gas chamber with 20% CO_2_ for 5 min prior to SI testing.

One week after the SI tests, rats were optically stimulated for 5 min during low/high (7.5/20%) CO_2_ exposure. We used the panicogenic challenge at low (7.5%) and high (20%) CO_2_ concentrations as they were previously shown to induce anxiety-like behavior, increased blood pressure, and hyperventilation [14, 15]. In short, rats were submitted to a constant infusion of atmospheric air (<1% CO_2_/21% O_2_/79% N_2_) in a Plexiglas chamber until animals (1) acclimated to the noise and stopped exploring the chamber and (2) showed stable cardiovascular parameters (baseline) for at least 5 min. Next, animals were challenged with a premixed experimental normoxic (21% O_2_), a hypercapnic gas infusion that contained either 7.5% or 20% of CO_2_ (71.5% or 59% N_2_) for an additional 5 min, followed by 40 min interval of atmospheric air. Each rat received both CO_2_ concentrations one week apart in a counterbalanced manner. Lastly, rats were submitted to the RTPP/A test (see “Experiment 1”) 1 week after the CO_2_ challenge. Ninety minutes prior to euthanasia, rats were photostimulated during a 20% CO_2_ exposure.

### Experiments 4 and 5—Chronic Loss-of-Function: Lesioning Effects of the PFA-projecting serotonergic neurons with SAP toxin on anxiety-, panic- and fear-associated behaviors

*Experiment 4*: We utilized SERT-SAP toxin to specifically lesion the PFA-projecting serotonergic system. Adult Sprague Dawley rats (300–350 g) received either SERT-SAP or the control IgG-SAP (Kit-23, Advanced Targeting Systems, San Diego, CA) via an injector (33 gauge, C311I, Plastics One, Roanoke, VA) that was connected to bilateral guide-cannulas (26 gauge, C311G, Plastics One) implanted into the PFA (AP: −3.0; ML: ±2.93; DV: −8.5, 15° oblique to the midsagittal plane) as previously described by us [41] and others [48]. Rats also were implanted with DSI probes as described above for cardiovascular assessments. In order to confirm that we selectively lesioned the PFA-projecting serotonergic neurons, IgG- and SERT-SAP-injected rats received bilateral injections of 100 nl of CTB (1% w/v in ACSF, List Biological Laboratories, Campbell, CA) over 5 min into the PFA three weeks prior to euthanasia as previously described by us [41].

*Experiment 5*: Rats from experiment 4 were first tested in SI, followed by elevated T-maze (ETM) testing as described [49]. Briefly, animals were exposed 3 times to the distal ends of the closed and open arms with a cutoff value set at 300 s and ITIs of 30 s. The time to leave the closed and open arms was determined as inhibitory avoidance and escape behaviors, respectively, and were manually scored by an experimenter blind to the experimental groups. Immediately after the last exposure to the open arm, the animals were tested in the open field for general locomotor activity. Two days later, rats were exposed to low/high (7.5/20%) CO_2_ challenges as described previously.

Rats were exposed to the fear conditioning paradigm one week after the CO_2_ challenge, as described previously [41, 50]. Briefly, on day 1—habituation session—rats were exposed for 10 min to the conditioning box with a grid floor that was connected to a scrambled shock generator (Ugo Basile, Gemonio, Italy). The conditioning box was placed in a larger sound-attenuated chamber (background noise and light set at 60 dB and 15-lux) during all sessions. On day 2—acquisition session—rats were submitted to 5 trials consisting of a 20 s, 4 kHz, 80 dB tone [conditioned stimulus (CS)] that co-terminated with a 0.5 s, 0.8 mA single footshock [unconditioned stimulus (US)] with inter-trial intervals (ITIs) of 100 s. On day 3—recall/extinction session—rats were exposed to 20 CS. Total time freezing during the CS were manually scored by an experimenter blind to the SAP treatments.

### General methods for Experiments 1–5

#### Immunohistochemistry

Brain tissue preparation, slicing, immunohistochemistry, and immunofluorescence procedures were carried out as described previously [41]. We performed the following reactions: (1) double staining for cFos and TPH (DR/MR) or cFos and OX (PFA) to identify brain regions and neurochemical systems involved in responses to optical stimulation; (2) single SERT staining (PFA and amygdala-control site) and double TPH/CTB immunofluorescence (DR/MR) to respectively confirm lesioning of local serotonergic fibers and their associated cell bodies; and (3) double SERT/eYFP (PFA) and TPH/eYFP (DR/MR) immunofluorescence to confirm colocalization between the serotonergic system and the viral constructs in the local fibers and cell bodies, respectively.

We used the following primary antibodies: goat anti-CTB (cat. no. 703, List Biological Laboratories; 1:1000–2000), rabbit anti-SERT (cat. no. 24330, ImmunoStar; 1:500–1000), rabbit anti-cFos (cat. no. SC52, Santa Cruz Biotechnology; 1:500), mouse anti-TPH (cat. no. T0678, Sigma-Aldrich; 1:300–1000), and rabbit anti-OX (cat. no. H-003-30, Phoenix Pharmaceuticals; 1:12,000).

#### Photography and densitometry of SERT^+^ fibers and quantification of cFos^+^ cells

Photomicrographs were obtained with a Leica DMLB microscope or a Nikon A1R+ confocal microscope. Densitometry analyses were done on grayscale inverted photographs from PFA or amygdala (control site) using Adobe Photoshop version 16. The IgG-SAP group mean represented 100% which was compared to SERT-SAP values. The numbers of cFos^+^ cells were counted in the entire field of view at 400x magnification in the PFA, MR, and divisions of the DR. All cell counts were done by a person blind to the experimental procedures.

#### Statistical analysis

The following dependent variables were analyzed using a two-tailed independent Student’s *t*-test (densitometry, SI, and cell counts). The colocalization analysis between SERT and ChR2-eYFP was determined using Pearson’s correlation coefficient with Nikon NIS Elements software. Conditioned fear (i.e., freezing) and place preference/avoidance responses were analyzed using a two-way analysis of variance (ANOVA) with saporin or AAV injections as between-subjects factors and time or session as the within-subjects factor. In the presence of significant main effects, between-subjects post hoc tests were conducted using Fisher’s least significant difference (LSD) tests. Statistical significance was accepted with *p* ≤ 0.05. All statistical analyses and graphs were carried out and generated using GraphPad Prism 7.04 for Windows (GraphPad Software Inc., San Diego, CA).

## Results

### Experiment 1: Effects of optogenetic stimulation of CaMKIIa-expressing neurons in the PFA

**Fig. 1:**
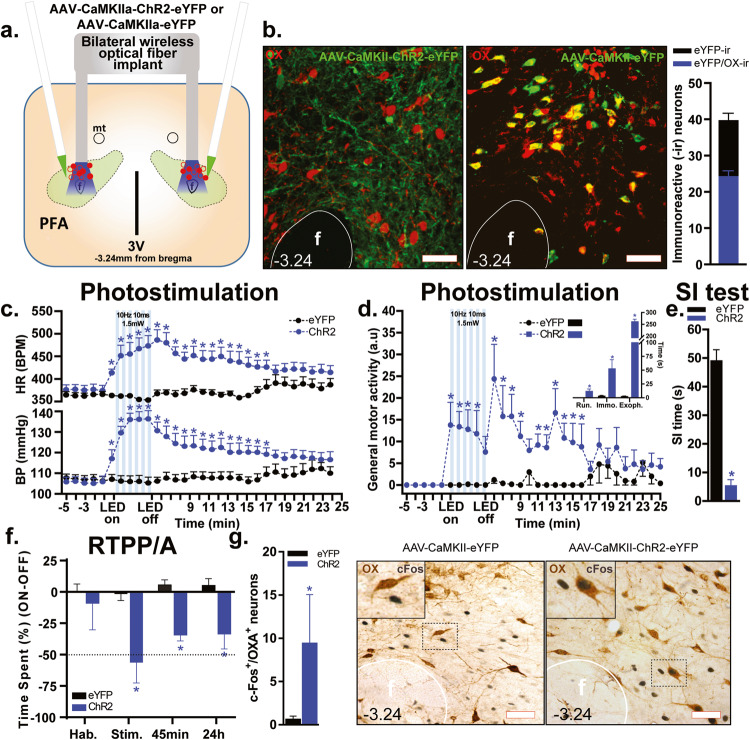
Wireless LED stimulation of glutamatergic cell bodies in the perifornical hypothalamic area (PFA) elicits escape behavior associated with cardiovascular excitation in rats that resembles a panic attack in humans. **a** Schematic illustration of AAV5-CaMKIIa-ChR2-eYFP or its control AAV-CaMKIIa-eYFP bilateral injection into the PFA and implantation of an optical fiber cannula in the PFA. Optic fiber placements (red circles: ChR2, filled; eYFP, hollow) ranged from −2.76 mm to −3.24 mm relative to bregma (shown collapsed in this schematic for conciseness purposes). **b** Representative confocal images showing ChR2-eYFP expression in fibers (ChR2, green, left photomicrograph) and somas (control, green, right photomicrograph) of orexin A peptide (OX)-expressing neurons (red, Alexa 640) in the PFA. The total average number of cell bodies co-expressing OX and eYFP (bar graph, right panel, Pearson correlation coefficient 0.63, *p* < 0.01). **c** Graph showing that light stimulation of PFA CaMKIIa-expressing cell bodies in ChR2-expressing rats induced a significant increase in blood pressure (BP, bottom graph), heart rate (HR, top graph, beats per minute, BPM), **d** general motor activity (line graph, *p < 0.01, between subjects differences using a Fisher’s LSD post hoc test) and its qualitative behavioral responses (bar graph inset, two-tailed, unpaired Student’s *t*-test, *p < 0.01), i.e., running (Run.), immobility (Immo.) and exophthalmos (Exoph.) during photostimulation (10 Hz, 10 ms, 1.5 mW, LED on-LED off, 5 min). **e** Bar graph demonstrating that ChR2-expressing rats had significantly less social interaction (SI) time during photostimulation of the PFA in the SI test (two-tailed, unpaired Student’s *t*-test, *p < 0.0001). **f** Bar graph showing that ChR2-expressing rats had a significantly lower difference score (percentage time spent on the stimulation side minus percentage time spent in non-stimulation side, *p < 0.05, between subjects differences using a Fisher’s LSD post hoc test) compared to the control group during light stimulation (Stim.), 45 min, and 24 h sessions, but not during habituation (Hab.) session in the real-time place preference/avoidance (RTPP/A) test. **g** Bar graph showing that ChR2-expressing rats (right panel) had significantly higher activation of the immediate early gene—cFos in the OX^+^ neurons (Mann–Whitney test, *p = 0.007) compared to the control group (left panel). Numbers on the bottom left of photomicrographs represent the distance (in mm) from bregma. Scale bars (bottom right): 200 μm. Abbreviations: 3V third ventricle, f fornix.

Viral expression of eYFP was observed in neuronal somas (AAV-CaMKIIa-eYFP) and fibers (AAV-CaMKIIa-ChR2-eYFP) within the PFA (see hit map of optical fibers in Fig. 1a and viral expression in Fig. 1b, *n* = 5/group). Approximately 62% of eYFP-immunoreactive (-ir) soma in the PFA also co-expressed OX (total average number of neurons in Fig. 1b, Pearson correlation coefficient 0.63, *p* < 0.01). Photostimulation in the PFA of ChR2-injected rats, but not control virus-injected rats, led to robust increases in heart rate (HR, group × time interaction *F*_(30,240)_ = 6.47, *p* < 0.0001, Fig. 1c, top graph), blood pressure (BP, group × time interaction *F*_(30,240)_ = 14.26, *p* < 0.0001, Fig. 1c, bottom graph), and general motor activity (group × time interaction *F*_(30,240)_ = 3.37, *p* < 0.0001, Fig. 1d), as well as qualitatively analyzed as aversive behaviors [i.e., long periods of exophthalmos (*p* < 0.0001) and immobility (*p* < 0.0001) followed by running (*p* = 0.007), Fig. 1d, inset]. Optogenetic stimulation of ChR2-injected rats also increased anxiety-like behavior as measured by a significant decrease in SI time compared to rats injected with the control virus (two-tailed, Student’s *t*-test, *p* < 0.0001, Fig. 1e) without promoting aggressive behavior. To determine if ChR2 stimulation would induce aversive behavior, rats were tested in the RTPP/A test, where one side of the chamber was paired with optical stimulation. Rats showed no side preference during the habitation session. However, the percent difference in time spent in the stimulation chamber was significantly reduced in the ChR2-injected rats when compared with the control group (group effect *F*_(1,8)_ = 22.91, *p* = 0.001) during the stimulation session (*p* = 0.001), and in the sessions 45 min (*p* = 0.01) and 24 h (*p* = 0.01, Fig. 1f) thereafter. Lastly, optical stimulation 90 min prior to euthanasia induced a marked increase of cFos in OX-ir neurons in ChR2-rats compared to the control counterparts (Mann–Whitney test, *p* = 0.007, Fig. 1g).

### Experiment 2: Anatomical confirmation of PFA-projecting serotonergic systems with CTB

**Fig. 2:**
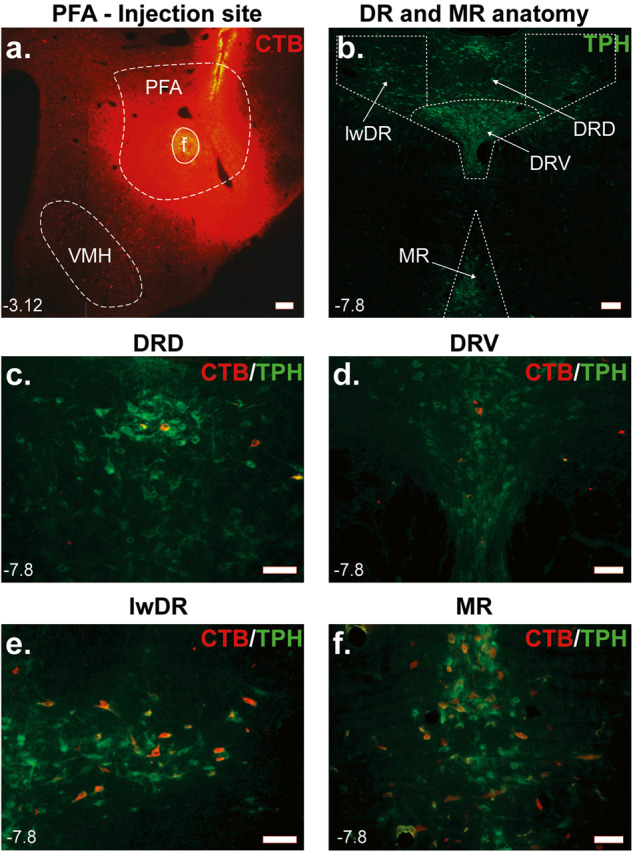
Retrograde tracing images from the perifornical hypothalamic area (PFA) using Cholera toxin B subunit (CTB). **a** Injection site illustrating CTB injection (Alexa 567, red) into the PFA. **b** Topography of tryptophan hydroxylase (TPH, Alexa 488, green) immunoreactive serotonergic neurons in the midbrain raphe nuclei [i.e., median raphe (MR) nucleus and dorsal (DRD), ventral (DRV) and lateral wing (lwDR) divisions of the dorsal raphe (DR) nucleus]. **c**–**f** Images showing TPH and CTB immunostainings in the MR and divisions of the DR. Note that most PFA-projecting serotonergic neurons originate from **e** lwDR and **f** MR, but not from **c** DRD and **d** DRV. Numbers on the bottom left of photomicrographs represent the distance (in mm) from bregma. Scale bars (bottom right): 200 μm. Abbreviations: f fornix, VMH ventromedial hypothalamus.

The PFA receives selective serotonergic inputs from distinct regions within the DR and MR. Rats (*n* = 7) injected with the retrograde tracer CTB into the PFA (Fig. 2a) had CTB-ir neurons in the serotonergic nuclei (see topography of midbrain TPH-ir neurons in Fig. 2b), both the DR and MR (Fig. 2c–f). Ninety-six percent of these CTB-labeled soma were serotonergic (i.e., colocalized with TPH-ir neurons). Within the serotonergic nuclei, many of the CTB-labeled neurons were in the MR and the lateral wings subdivision of the DR (lwDR), which contained 29% and 63% of the total CTB-labeled neurons, respectively, while the dorsal (DRD) and ventral (DRV) subdivisions of the DR only contained 3%, and 5% of these labeled neurons, respectively. A small amount of CTB/TPH-labeled neurons were also observed in the caudal part of the dorsal raphe nucleus (DRC, not shown), where CTB injections spread closer to the meninges.

### Experiment 3: Effects of optogenetic stimulation of DR/MR fibers in the PFA on physiological and behavioral responses to anxiety- and panic-associated challenges

**Fig. 3:**
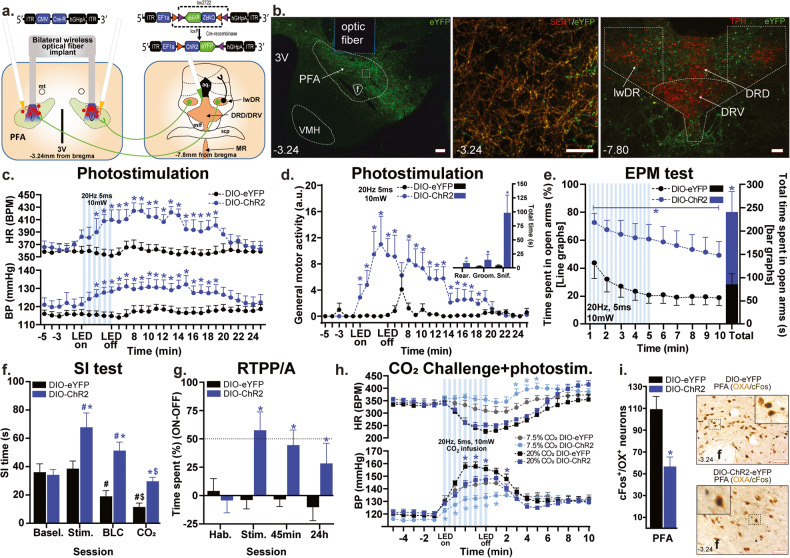
In the gain-of-function study, wireless LED stimulation of perifornical-hypothalamic-area-(PFA)-projecting serotonergic fibers reduced anxiety- and panic-associated responses and promoted exploration. **a** Schematic illustration of rats bilaterally injected with AAV5-DIO-ChR2-eYFP or AAV5-DIO-eYFP (not illustrated) into the dorsal raphe nucleus (DR) and CAV-CMV-Cre into the PFA, and photostimulation with bilateral wireless optical fiber implants in the PFA. Optic fiber placements (red circles: ChR2, filled; eYFP, hollow) ranged from −2.76 mm to −3.24 mm relative to bregma (shown collapsed in this schematic for conciseness purposes). **b** Representative confocal images showing: *left—*ChR2-eYFP expression of dorsal raphe (DR) fibers (green) in the PFA region, *middle—*SERT immunostaining (SERT, red, Alexa 640) of ChR2-eYFP raphe to PFA inputs confirmed that the majority of raphe to PFA projections are serotoninergic (Pearson correlation coefficient 0.92, *p* < 0.001); *right—*ChR2-eYFP expression (green) in tryptophan hydroxylase (TPH)^+^ neurons (red, Alexa 640) in the lateral wings (lwDR), the dorsal (DRD) and ventral (DRV) DR. Median raphe nucleus (MR) is not shown. Numbers indicate distance, in mm, from bregma according to rat brain atlas [38]. Optogenetic stimulation (20 Hz, 5 ms, 10 mW, LED on-LED off, 5 min) of lwDR/MR → PFA:ChR2 expressing fibers induced a significant **c** increase in blood pressure (BP, bottom graph), heart rate (HR, top graph, beats per minute, BPM) and **d** general motor activity and behavioral responses (inset bar graph). **e** Optogenetic stimulation of lwDR/MR → PFA:ChR2-expressing fibers in the PFA increased open arm exploration during (1–5 min) and post (6–10 min, line graphs, left *y*-axis) stimulation in the elevate plus-maze (EPM) test (see total time, in s, of open arm exploration in bar graphs and right *y-*axis). Compared to controls, photostimulation of lwDR/MR → PFA:ChR2 expressing fibers led to **f** increase in social interaction (SI) time during stimulation (Stim.), bright-light challenge (BLC), and CO_2_ sessions [symbols are: *comparison between groups; ^#^within groups compared to baseline (Basel.) or ^$^to stimulation (Stim.)]; **g** increase of the difference score (percentage time spent in stimulation side minus percentage time spent in non-stimulation side, *p < 0.05, between subjects differences using a Fisher’s LSD post hoc test) compared to the control group during light stimulation (Stim.), 45 min, and 24 h sessions, but not during habituation (Hab.) session in the real-time place preference/avoidance (RTPP/A) test; **h** attenuation of CO_2_-induced increases in BP (bottom graph) and HR (top graph) in the CO_2_ challenge + photostimulation (photostim.) test; and **i** attenuation of cFos responses in orexin (OX)^+^ neurons in the PFA. Graphs in (**c**–**h**) are two-way ANOVA followed by Fisher’s LSD post hoc test, whereas bar graphs in (**d**, **e**, **i**) are unpaired Student’s *t*-test. * and # are *p* < 0.05. Numbers on the bottom left of photomicrographs represent the distance (in mm) from bregma. Scale bars (bottom right): 200 μm. Abbreviations: 3V third ventricle, f fornix, mt mammillothalamic tract, OX orexin A peptide, VMH ventromedial hypothalamus.

Using a two-virus approach, we injected the retrogradely trafficked CAV-CMV-Cre bilaterally into the PFA and either a control (AAV-EF1a-DIO-eYFP) or active ChR2 (AAV-EF1a-DIO-ChR2-eYFP) Cre-dependent viruses into the DR. We used this intersectional approach to selectively evaluate the role of DR/MR fibers in anxiety and panic (Fig. 3a). In the ChR2 group, we confirmed that the majority of eYFP^+^ fibers in the PFA (Fig. 3b, photo on the left) co-localized with SERT signal (Fig. 3b, photo in the middle, Pearson correlation coefficient 0.92, *p* < 0.001). Neuronal expression of eYFP in the DR and MR (Fig. 3b, photo on the right) followed a similar lwDR/MR topography observed in the CTB-labeled neurons in “Experiment 2”.

While HR and BP did not differ between the viral constructs during baseline, wireless LED stimulation of ChR2-expressing fibers in the PFA led to a mild increase in BP (group effect, *F*_(1,17)_ = 4.2, *p* = 0.05), HR (*F*_(1,17)_ = 12.83, *p* = 0.002, Fig. 3c), and locomotion [i.e., general exploratory/self-directed behaviors: sniffing (*p* = 0.008), rearing (*p* = 0.02), and grooming (*p* = 0.02), *n* = 10 DIO-eYFP, *n* = 9 DIO-ChR2, Fig. 3d].

Next, photostimulation of serotonergic fibers in the PFA paired with open-arm exploration in the EPM of the ChR2 group greatly increased the total time spent in the open arm when compared to the control. This effect persisted for the subsequent 5 min in the absence of photostimulation (group effect *F*_(1,14)_ = 12.34, *p* = 0.003, Fig. 3e).

The SI testing was carried out in four different sessions tested throughout four consecutive days: baseline (*n* = 9 eYFP, *n* = 10 ChR2), optical stimulation (*n* = 9 per group), bright light challenge (*n* = 9/group), and 20% CO_2_ challenge (*n* = 7/group). In the ChR2 group, light stimulation of serotonergic fibers in the PFA increased total SI time during all sessions compared to baseline and the control virus group (ChR2 effect: *F*_(1,61)_ = 23.8, *p* < 0.0001, session effect: *F*_(3,61)_ = 10.42, *p* < 0.0001 and ChR2 × session interaction: *F*_(3,61)_ = 4.08, *p* = 0.01, Fig. 3f).

Light stimulation in the RTPP/A test led to a strong place preference behavior in the ChR2-expressing group [group × session (*F*_(3,39)_ = 3.75, *p* = 0.01)] during optical stimulation (*p* = 0.001), 45 min (*p* = 0.01), and 24 h (*p* = 0.03) sessions (*n* = 7 DIO-eYFP, *n* = 8 DIO-ChR2; Fig. 3g).

Lastly, optical stimulation of the serotonergic fibers in the PFA attenuated CO_2_-induced (7.5%, *n* = 9 DIO-eYFP, *n* = 8 DIO-ChR2; and 20%, *n* = 9/group) bradycardia (7.5% only, group × time interaction *F*_(19,285)_ = 2.25, *p* = 0.0024 Fig. 3h, top graph) and increases in BP (group × time interaction *F*_(19,285)_ = 2.61, *p* = 0.0003 and *F*_(19,304)_ = 2.61, *p* = 0.0003, respectively, Fig. 3h, bottom graph). Importantly, there was a reduction of cFos expression in OX-ir neurons in the PFA of ChR2 animals 90 min post-stimulation of the serotonergic fibers when compared to the control virus (*t*_(8)_ = 3.6, *p* = 0.006, *n* = 5 each, Fig. 3i).

### Experiment 4: Effects of SERT-SAP injection into the PFA on local SERT fibers and their associated cell bodies in the lwDR and MR

**Fig. 4:**
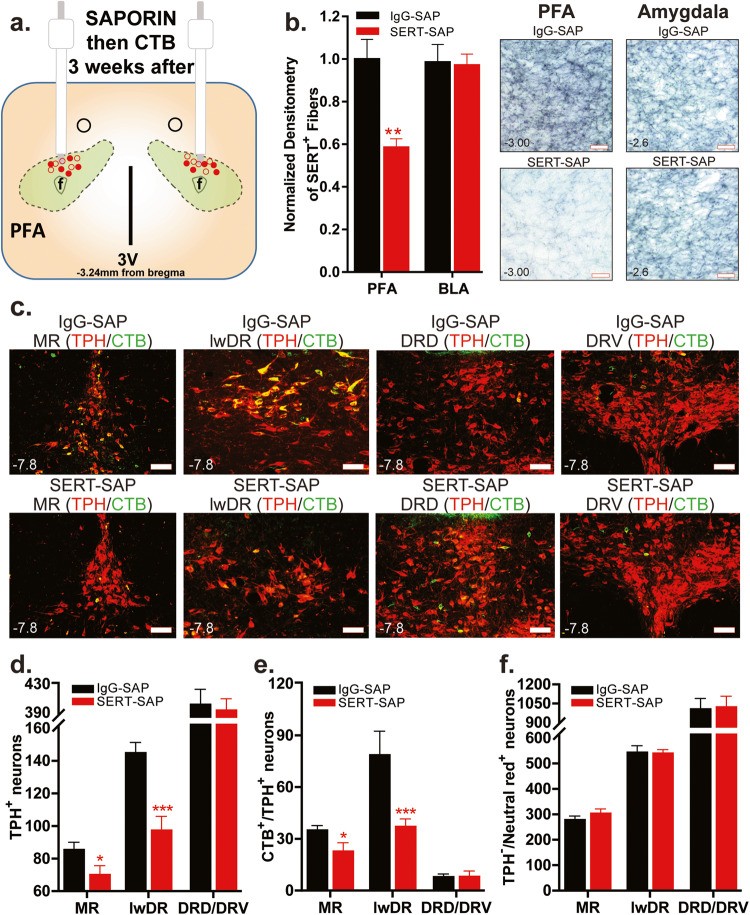
Injection of saporin (SAP) toxin conjugated to the serotonin transporter antibody (SERT-SAP) lesions local perifornical hypothalamic area (PFA) SERT^+^ fibers and their associated tryptophan hydroxylase (TPH)^+^ cell bodies in the median (MR) and lateral wings (lwDR) division of the dorsal raphe (DR). **a** Schematic illustration of the bilateral injection of SERT-SAP or its control IgG-SAP followed by injection of the retrograde tracer Cholera toxin b subunit (CTB) into the PFA. Cannula placements (red circles: SERT-SAP, filled; IgG-SAP, hollow) ranged from −2.76 mm to −3.24 mm relative to bregma (shown collapsed in this schematic for conciseness purposes). **b** SERT-SAP injected rats had significantly reduced (two-tailed, unpaired Student’s *t*-test, *p = 0.001) expression of SERT^+^ fibers in the PFA (left lower panel photograph) than IgG-SAP rats (left upper panel photograph), but not in the basolateral amygdala (BLA, right panel photographs). **c** SERT-SAP injected rats (bottom row) had decreased total number of TPH^+^ [red, Alexa 640, bar graphs in (**d**)] and CTB^+^/TPH^+^ [CTB green, Alexa 488, bar graphs in (**e**)] neurons in the lateral wings (lwDR) DR and MR, but not in the dorsal (DRD)/ventral (DRV) DR when compared to IgG-SAP group (top row). See the topography of MR and divisions of DR in Fig. 2b. **f** Using Neutral Red staining, we have confirmed that SERT-SAP injections did not reduce the total number of non-TPH neurons in any of the DR and MR subregions [two-tailed, unpaired Student’s *t*-test, *p < 0.05, and ***p < 0.0005 for (**d**–**f**)]. Numbers on the bottom left of photomicrographs represent the distance (in mm) from bregma. Scale bars (bottom right): 200 μm. Abbreviations: 3V third ventricle, Aq. aqueduct, f fornix.

SERT-SAP injection in the PFA (Fig. 4a) significantly reduced the density of local SERT-positive fibers in the PFA by approximately 42% (two-tailed, unpaired Student’s *t*-test, *p* = 0.001) but did not change the density of SERT-positive fibers in the amygdala (control site, *n* = 7 each, Fig. 4b), which receives sparse fibers from PFA-projecting 5-HT neurons [30]. In control, IgG-SAP rats, bilateral injections of CTB into the PFA led to CTB expression in many TPH^+^ neurons in the MR and lwDR, but few in DRD and DRV (Fig. 4c). SERT-SAP injections into the PFA also respectively reduced the total number of single-TPH^+^ (Fig. 4d) and double-CTB^+^/TPH^+^ (Fig. 4e) neurons in the MR (*p* = 0.03; *p* = 0.02) and lwDR (*p* = 0.0004; *p* = 0.01), but not in the DRD or DRV (*n* = 7 IgG-SAP, 6 SERT-SAP; one animal excluded due to poor TPH staining). SERT-SAP injections into the PFA did not lesion non-TPH neurons in the DR or MR (neutral red^+^ cells, Fig. 4f).

### Experiment 5: Effects of selective lesion of serotonergic fibers in the PFA on physiology and panic-like behaviors

**Fig. 5:**
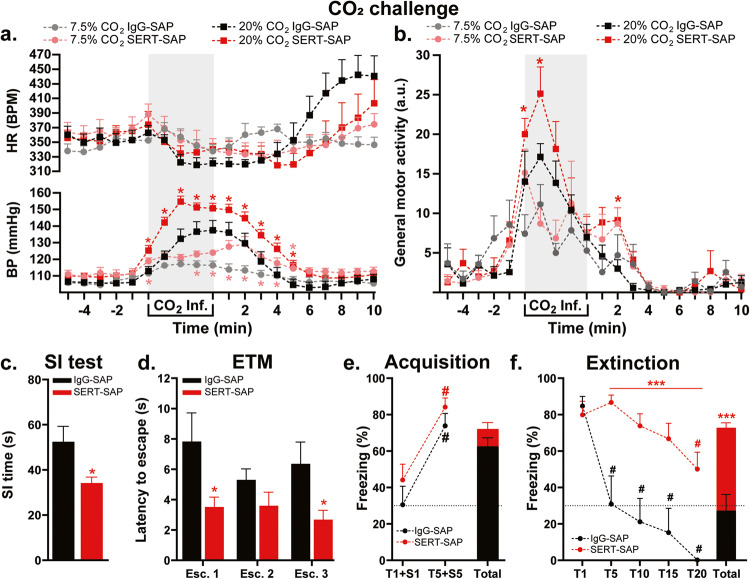
In the loss-of-function study, injection of saporin (SAP) toxin conjugated to the serotonin transporter antibody (SERT-SAP) into the perifornical hypothalamic area (PFA) promoted anxiety-, fear-, and panic-associated responses. SERT-SAP injected rats showed **a** greater increase in blood pressure (BP, bottom graph) and **b** general motor activity in the CO_2_ challenge; **c** reduction of social interaction (SI) time in the SI test; **d** decreased latency to escape the open arm in the elevated T-maze (ETM); **e** normal acquisition of freezing response to tone (T) + shock (S) pairings; and **f** greater freezing response and also delayed extinction of fear memories (*between groups; ^#^compared to T1 within groups). Note the hypercapnia-induced decrease in heart rate (HR, top graph, beats per minute, BPM) in both SAP groups compared to baseline (**a**). Part (**c**) and bar graphs in (**e**) and (**f**) are two-tailed, unpaired Student’s t test; bar graph in (**d**) and line graphs (**a**, **b**) and (**e**, **f**) are two-way ANOVA followed by Fisher’s LSD post hoc test. *p < 0.05; ***p < 0.0005; ^#^*p* < 0.05. Abbreviations: Atm atmospheric, Esc. escape response, Inf. CO_2_ infusion.

While the HR response did not differ between treatments in either CO_2_ concentration, there was hypercapnia-induced bradycardia (time effect *F*_(19,228)_ = 2.005, *p* = 0.0091 and *F*_(19,228)_ = 10.91, *p* < 0.0001, 7.5% and 20%, respectively, Fig. 5a, top graph). In SERT-SAP lesioned rats, compared to control rats, low CO_2_ (7.5%) exposure elicited moderate increases in BP (treatment effect *F*_(1,12)_ = 11.01, *p* = 0.006), whereas higher CO_2_ (20%) concentration induced robust elevation in both BP (treatment effect *F*_(1,12)_ = 7.62, *p* = 0.017 and treatment × time interaction *F*_(19,228)_ = 3.65, *p* < 0.0001, Fig. 5a, bottom graph) and locomotor activity (treatment effect *F*_(1,12)_ = 8.54, *p* = 0.01, Fig. 5b). SERT-specific lesioning in the PFA led to reduction of total SI time (*p* = 0.02, *n* = 7 each, Fig. 5c) and decrease of the latency to escape from the open arm in the ETM test, compared to control group (treatment effect *F*_(1.11)_ = 7.16, *p* = 0.021, *n* = 7 IgG-SAP, *n* = 6 SERT-SAP; one animal excluded due repeated falls from ETM; Fig. 5d). Pre-treatment with SERT-SAP did not have any effects on avoidance in the ETM and OF test (not shown). Lastly, selective lesioning of PFA serotoninergic fibers did not affect fear acquisition (Fig. 5e), but did increase overall freezing responses during fear extinction, compared to controls (treatment effect *F*_(1,12)_ = 23.72, *p* = 0.0004 and treatment × time interaction *F*_(19,228)_ = 2.69, *p* = 0.0003, Fig. 5f).

## Discussion

With the majority of the eYFP signal in the PFA being colocalized with OX neurons, photostimulation of ChR2 in these PFA neurons led to robust expression of behavioral and physiological panic-associated responses and an increase in cellular cFos responses in OX-ir neurons compared to control virus rats. These responses were similar to those described in the literature utilizing less selective pharmacological [7, 51–53] and electrical stimulation [23, 54] of the PFA in rats. Wireless LED stimulation of these ChR2-expressing neurons in PFA also induced conditioned place avoidance, lasting at least 24 h. These effects indicate the aversive nature of a subpopulation of CaMKIIa-positive neurons in the PFA, most of which were OX, and indicate the capacity of this region to organize some forms of emotional learning canonically seen in fear conditioning experiments [55, 56]. Collectively, optogenetic stimulation of the CaMKIIa PFA system promotes orchestrated behaviors accompanied by physiological responses that are components of the “fight-or-flight” repertoire, thus reinforcing its role as one of the putative brain regions underlying panic response. Even though most neurons expressing the CaMKIIa viral construct in experiment 1 were also OX, the construct was not designed to selectively target the OX system. Therefore, it cannot be ruled out that at part of the responses to photostimulation in this experiment were a result of stimulation of a separate population of ChR2-positive neurons that do not express OX or a combination of the entire ChR2-positive neurons within the PFA.

Serotonin has been linked to reducing panic response and panic-like behaviors evoked by local electrical stimulation [23] or GABAergic disinhibition with bicuculine [25] in the PFA. Here, we report that the likely source of endogenous serotonergic inputs to the PFA arises from the lwDR and MR. Following retrograde tracer CTB injections into the PFA, nearly all of the raphe serotonergic neurons containing CTB originated in the lwDR and MR, consistent with previous tracing studies [29, 30]. However, CTB/TPH-ir neurons were also seen in the DRC in fewer cases where CTB injections spread closer to the meninges, similarly as described by others [57].

We then used an intersectional genetics approach to selectively induce the expression of ChR2 in the lwDR and MR neurons projecting to the PFA. Using SERT immunohistochemistry, we have also confirmed that these lwDR/MR to PFA projections are serotonergic. Photostimulation of lwDR/MR → PFA:ChR2 fibers attenuated the suffocation-associated stressor-induced increases in pressor responses and reductions in SI time, as well as decreased the number of cFos responses in OX neurons within the PFA. Similarly to our previous observations [15, 58] and others [59], both respiratory challenges promoted the hypercapnic-induced bradycardia response [60]. This suffocation-associated stressor (5 min infusion of normoxic 20% CO_2_) elicits core symptoms of a panic attack in humans (e.g., catastrophic fear accompanied by cardiovascular and thermoregulatory responses) [61] and has predictive validity since fluoxetine and alprazolam, two clinically relevant panicolytic drugs utilized to treat patients with recurrent panic attacks, attenuate panic-related behavioral [62] and physiological responses [14, 63] elicited by 20% CO_2_ inhalation in rodents. Collectively, these data support the hypothesis that PFA-projecting serotonergic fibers originating from the lwDR/MR inhibit innate panic behaviors and anxiety avoidance [32, 33].

In addition to reducing panic-like responses, under baseline conditions, light stimulation of ChR2-expressing fibers in the PFA induced a mild excitatory cardiovascular response, and rats displayed investigation-driven behaviors, such as increased exploration, accompanied by sniffing and occasional rearing, which may have contributed to the cardioexcitation. It is noteworthy to mention that the repertoire of the behavior responses to the light-induced activation of lwDR/MR → PFA fibers was qualitatively different from the group of animals where we photostimulated cell bodies of CaMKIIa neurons in the PFA. In agreement with these results, 5-HT release in the hypothalamus has been linked to the control of wakefulness responses in different situations [64–66]. Moreover, light stimulation of ChR2-expressing lwDRN/MR PFA fibers also increased time spent in the open arm, increased SI time, and induced conditioned place preference, an effect observed up to 24 h post-conditioning. Collectively, all these behavioral data suggest that activation of the serotonergic inputs from the lwDRN/MR to the PFA exerts an anxiolytic effect.

Our observations following photostimulation of lwDR/MR → PFA:ChR2 fibers suggest a serotonergic mechanism, and the panic-inhibiting observations are in line with previous publications demonstrating expression of the 5-HT_1A_ receptor on PFA OX neurons [27, 28] and pre-treatment with a 5-HT_1A_ receptor antagonist blocks 5-HT inhibition of OX neurons [28]. Thus, it is possible that stimulation of the lwDR/MR → PFA fibers induces the release of 5-HT that directly inhibits OX neurons via the 5-HT_1A_ receptor, resulting in inhibition of these neurons and reducing panic-like behavior and physiological responses. Alternatively, stimulation of the lwDR/MR → PFA fibers may inhibit the OX neurons via a GABA mechanism. OX neurons within the PFA express GABA_A_ receptors and are disinhibited and inhibited by GABA_A_ receptor antagonists and agonizts, respectively [67]. Along those lines, in vitro bath application of 5-HT or photostimulation of 5-HT fibers expressing ChR2 in the PFA facilitates GABA_A_-mediated inhibitory tone to OX neurons [68]. Here it is likely that serotonin indirectly induces the GABA_A_-mediated inhibition of PFA-OX neurons via activation of GABAergic interneurons rather than direct release of GABA from the serotonergic terminals as very few (0.1–0.7%) of PFA-projecting serotonergic neurons in the DR and MR co-express GAD [69–71]. It remains unclear if the effects observed here by photostimulation are a direct result of only local 5-HT release [72–76] or if the optical stimulation also resulted in the release of 5-HT from collaterals via antidromic stimulation [77–80].

In our final experiments, we investigated the effect of the selective lesioning of lwDR/MR serotonergic projections on the PFA on panic and anxiety behaviors. SERT-SAP injections into the PFA reduced SERT-ir fibers in PFA and selectively lesioned serotonergic neurons of the lwDR and MR, but not in the DRD/DRV. It is important to note that no alterations in the density of SERT-ir fibers were observed in the basolateral amygdala (BLA); thus, it is unlikely that the results observed here were due to a loss of serotonergic terminals beyond those targeted by the injections of SERT-SAP in the PFA. This region receives fewer serotonergic inputs, if any, from the lwDR/MR [30] and, for this reason, was used as a negative control in this experiment. More importantly, selective lesions of the lwDR/MR → PFA serotonergic circuit increased innate anxiety- and panic-associated responses measured by reduced SI time decreased escape latency in the ETM, enhanced general locomotor activity, and increased pressor response to CO_2_ challenges. Lesioning of 5-HT fibers in the PFA produced panic-like effects similar to the panic-vulnerable state induced by chronic disinhibition of GABA synthesis in the PFA [31, 81, 82].

Lesioning of the PFA-projecting serotonergic neurons did not alter fear acquisition, but it did lead to fear extinction resistance. Although we did not see any significant reduction of 5-HT fibers in the amygdala, we cannot exclude the possibility that this region was involved in the alterations of freezing behavior observed in our experiments. This is because we [34] and others [83] have shown that OX neurons in the PFA project heavily to the amygdala and play a critical role in the consolidation and extinction of aversive memories [34–36] via OX1 receptors [35]. This role of PFA glutamatergic/orexinergic neurons in the context of the mechanisms of persistent fear was further explored by Molosh and colleagues [34], where they showed that light-induced activation of BLA-projecting neurons in the PFA leads to resistant extinction of fear memories and greater persistence of fear memories. Therefore, another possible explanation of our results of the fear conditioning experiment is that selective lesioning of serotoninergic inputs from the lwDR/MR induces disinhibition of PFA neurons that in turn, excite BLA neurons. However, future studies need to delve deeper into the functional interplay between 5-HT, OX, and glutamate in the PFA and amygdala.

Our data presented here contrasts with the view that 5-HT may have only a phasic role in the hypothalamus in the context of innate panic-like behavior [22, 23]. Our loss-of-function experiments likely reflect the fact that the ablation of the lwDR/MR → PFA serotonergic pathway causes a more profound elimination of 5-HT modulatory effects in the PFA than does pharmacological inhibition. It is possible that our circuit-targeted ablation may have lesioned other non-serotonergic populations at the raphe projecting sites. However, it is important to mention that the total number of neutral red-positive neurons in the various raphe nuclei remained unaltered following serotonergic ablation, thus weakening this possibility.

## Conclusions

It has been previously believed that central serotonergic systems are functionally homogeneous in regulating innate panic and conditioned fear. Recently, the idea that there are topographically organized subsets of 5-HT neurons with unique afferents, efferents, and functional properties has been proposed [20]. Based on the data presented here, we have identified a novel 5-HT sub-system that projects to and inhibits a panic-generating brain region that also influences conditioned fear responses. These data provide new insights into underlying mechanisms of panic and fear and how selective disruption of PFA-projecting 5-HT neurons located in the DR/MR can lead to a vulnerability to panic disorder [84–86] and highly comorbid phobias [87].

## Data Availability

The data that support the findings of this study are deposited at an Indiana University School of Medicine institutional account of LabArchives. The data are available from the corresponding authors upon reasonable request.

## References

[1] Hess WR, Brugger M (1943). Das subkortikale zentrum der affektiven abwehrreaktion. Helv Physiol Pharm Acta.

[2] Anderson JJ, DiMicco JA (1990). Effect of local inhibition of γ-aminobutyric acid uptake in the dorsomedial hypothalamus on extracellular levels of γ-aminobutyric acid and on stress-induced tachycardia: a study using microdialysis. J Pharm Exp Ther.

[3] Olds ME, Olds J (1962). Approach-escape interactions in rat brain. Am J Physiol Content.

[4] Di Scala G, Schmitt P, Karli P (1984). Flight induced by infusion of bicuculline methiodide into periventricular structures. Brain Res.

[5] Duan Y-F, Winters R, McCabe PM, Green EJ, Huang Y, Schneiderman N (1996). Behavioral characteristics of defense and vigilance reactions elicited by electrical stimulation of the hypothalamus in rabbits. Behav Brain Res.

[6] Markgraf CG, Winters RW, Liskowsky DR, McCabe PM, Green EJ, Schneiderman N (1991). Hypothalamic, midbrain and bulbar areas involved in the defense reaction in rabbits. Physiol Behav.

[7] Shekhar A, DiMicco JA (1987). Defense reaction elicited by injection of GABA antagonists and synthesis inhibitors into the posterior hypothalamus in rats. Neuropharmacology.

[8] Soltis RP, DiMicco JA (1992). Hypothalamic excitatory amino acid receptors mediate stress-induced tachycardia in rats. Am J Physiol.

[9] Shekhar A, Hingtgen JN, DiMicco JA (1990). GABA receptors in the posterior hypothalamus regulate experimental anxiety in rats. Brain Res.

[10] Schoenen J, Di Clemente L, Vandenheede M, Fumal A, De Pasqua V, Mouchamps M (2005). Hypothalamic stimulation in chronic cluster headache: a pilot study of efficacy and mode of action. Brain.

[11] Rasche D, Foethke D, Gliemroth J, Tronnier VM (2006). Deep brain stimulation in the posterior hypothalamus for chronic cluster headache. Case report and review of the literature. Der Schmerz.

[12] Peyron C, Tighe DK, van den Pol AN, de Lecea L, Heller HC, Sutcliffe JG (1998). Neurons containing hypocretin (orexin) project to multiple neuronal systems. J Neurosci.

[13] Thannickal TC, Moore RY, Nienhuis R, Ramanathan L, Gulyani S, Aldrich M (2000). Reduced number of hypocretin neurons in human narcolepsy. Neuron.

[14] Johnson PL, Federici LM, Fitz SD, Renger JJ, Shireman B, Winrow CJ (2015). Orexin 1 and 2 receptor involvement in CO2-induced panic-associated behavior and autonomic responses. Depress Anxiety.

[15] Johnson PL, Samuels BC, Fitz SD, Lightman SL, Lowry CA, Shekhar A (2012). Activation of the orexin 1 receptor is a critical component of CO2-mediated anxiety and hypertension but not bradycardia. Neuropsychopharmacology.

[16] Johnson PL, Truitt W, Fitz SD, Minick PE, Dietrich A, Sanghani S (2010). A key role for orexin in panic anxiety. Nat Med.

[17] Bonaventure P, Dugovic C, Shireman B, Preville C, Yun S, Lord B (2017). Evaluation of JNJ-54717793 a novel brain penetrant selective orexin 1 receptor antagonist in two rat models of panic attack provocation. Front Pharm.

[18] Salvadore G, Bonaventure P, Shekhar A, Johnson PL, Lord B, Shireman BT (2020). Translational evaluation of novel selective orexin-1 receptor antagonist JNJ-61393215 in an experimental model for panic in rodents and humans. Transl Psychiatry.

[19] Nakamura M, Nagamine T (2017). Neuroendocrine autonomic, and metabolic responses to an orexin antagonist, suvorexant, in psychiatric patients with Insomnia. Innov Clin Neurosci.

[20] Hale MW, Shekhar A, Lowry CA (2012). Stress-related serotonergic systems: implications for symptomatology of anxiety and affective disorders. Cell Mol Neurobiol.

[21] Hood SD, Bell CJ, Argyropoulos SV, Nutt DJ (2016). Don’t panic. A guide to tryptophan depletion with disorder-specific anxiety provocation. J Psychopharmacol.

[22] Nascimento JOG, Kikuchi LS, de Bortoli VC, Zangrossi H, Viana MB (2014). Dorsomedial hypothalamus serotonin 1A receptors mediate a panic-related response in the elevated T-maze. Brain Res Bull.

[23] de Bortoli VC, Yamashita P, Zangrossi H (2013). 5-HT1A and 5-HT2A receptor control of a panic-like defensive response in the rat dorsomedial hypothalamic nucleus. J Psychopharmacol.

[24] Roncon CM, Yamashita PSDM, Frias AT, Audi EA, Graeff FG, Coimbra NC (2017). μ-Opioid and 5-HT1A receptors in the dorsomedial hypothalamus interact for the regulation of panic-related defensive responses. J Psychopharmacol.

[25] Biagioni AF, de Oliveira RC, de Oliveira R, da Silva JA, Anjos-Garcia Tdos, Roncon CM (2016). 5-Hydroxytryptamine 1A receptors in the dorsomedial hypothalamus connected to dorsal raphe nucleus inputs modulate defensive behaviours and mediate innate fear-induced antinociception. Eur Neuropsychopharmacol.

[26] Stamper CE, Hassell JE, Kapitz AJ, Renner KJ, Orchinik M, Lowry CA (2017). Activation of 5-HT 1A receptors in the rat dorsomedial hypothalamus inhibits stress-induced activation of the hypothalamic–pituitary–adrenal axis. Stress.

[27] Collin M, Bäckberg M, Onnestam K, Meister B (2002). 5-HT1A receptor immunoreactivity in hypothalamic neurons involved in body weight control. Neuroreport.

[28] Muraki Y, Yamanaka A, Tsujino N, Kilduff TS, Goto K, Sakurai T (2004). Serotonergic regulation of the orexin/hypocretin neurons through the 5-HT1A receptor. J Neurosci.

[29] Ljubic-Thibal V, Morin A, Diksic M, Hamel E (1999). Origin of the serotonergic innervation to the rat dorsolateral hypothalamus: retrograde transport of cholera toxin and upregulation of tryptophan hydroxylase mRNA expression following selective nerve terminals lesion. Synapse.

[30] Muzerelle A, Scotto-Lomassese S, Bernard JF, Soiza-Reilly M, Gaspar P (2016). Conditional anterograde tracing reveals distinct targeting of individual serotonin cell groups (B5–B9) to the forebrain and brainstem. Brain Struct Funct.

[31] Johnson P, Lowry C, Truitt W, Shekhar A (2008). Disruption of GABAergic tone in the dorsomedial hypothalamus attenuates responses in a subset of serotonergic neurons in the dorsal raphe nucleus following lactate-induced panic. J Psychopharmacol.

[32] Johnson PL, Lightman SL, Lowry CA (2004). A functional subset of serotonergic neurons in the rat ventrolateral periaqueductal gray implicated in the inhibition of sympathoexcitation and panic. Ann N Y Acad Sci.

[33] Johnson PL, Hollis JH, Moratalla R, Lightman SL, Lowry CA (2005). Acute hypercarbic gas exposure reveals functionally distinct subpopulations of serotonergic neurons in rats. J Psychopharmacol.

[34] Molosh AI, Dustrude ET, Lukkes JL, Fitz SD, Caliman IF, Abreu ARR (2018). Panic results in unique molecular and network changes in the amygdala that facilitate fear responses. Mol Psychiatry.

[35] Dustrude ET, Caliman IF, Bernabe CS, Fitz SD, Grafe LA, Bhatnagar S (2018). Orexin depolarizes central amygdala neurons via orexin receptor 1, phospholipase C and sodium-calcium exchanger and modulates conditioned fear. Front Neurosci.

[36] Flores Á, Valls-Comamala V, Costa G, Saravia R, Maldonado R, Berrendero F (2014). The hypocretin/orexin system mediates the extinction of fear memories. Neuropsychopharmacology.

[37] National Research Council. Guide for the Care and Use of Laboratory Animals: Eighth Edition. The National Academies Press: Washington, DC; 2011. 10.17226/12910.

[38] Paxinos G, Watson C. The rat brain in stereotaxic coordinates. Academic Press: San Diego, CA; 1998.

[39] Sanders SK, Morzorati SL, Shekhar A (1995). Priming of experimental anxiety by repeated subthreshold GABA blockade in the rat amygdala. Brain Res.

[40] File SE (1980). The use of social interaction as a method for detecting anxiolytic activity of chlordiazepoxide-like drugs. J Neurosci Methods.

[41] Bernabe CS, Caliman IF, Truitt WA, Molosh AI, Lowry CA, Hay-Schmidt A (2020). Using loss- and gain-of-function approaches to target amygdala-projecting serotonergic neurons in the dorsal raphe nucleus that enhance anxiety-related and conditioned fear behaviors. J Psychopharmacol.

[42] Soudais C, Laplace-Builhe C, Kissa K, Kremer EJ (2001). Preferential transduction of neurons by canine adenovirus vectors and their efficient retrograde transport in vivo. FASEB J.

[43] Junyent F, Kremer EJ (2015). CAV-2-why a canine virus is a neurobiologist’s best friend. Curr Opin Pharm.

[44] Schwarz LA, Miyamichi K, Gao XJ, Beier KT, Weissbourd B, DeLoach KE et al. Viral-genetic tracing of the input–output organization of a central noradrenaline circuit. Nature. 2015. 10.1038/nature14600. 10.1038/nature14600 PMC4587569 26131933

[45] Li Y, Zhong W, Wang D, Feng Q, Liu Z, Zhou J (2016). Serotonin neurons in the dorsal raphe nucleus encode reward signals. Nat Commun.

[46] Marcinkiewcz CA, Mazzone CM, D’Agostino G, Halladay LR, Hardaway JA, DiBerto JF (2016). Serotonin engages an anxiety and fear-promoting circuit in the extended amygdala. Nature.

[47] File SE, Hyde JRG (1978). Can social interaction be used to measure anxiety?. Br J Pharm.

[48] Nattie EE, Li A, Richerson G, Lappi DA (2004). Medullary serotonergic neurones and adjacent neurones that express neurokinin-1 receptors are both involved in chemoreception in vivo. J Physiol.

[49] Zangrossi H, Graeff FG (1997). Behavioral validation of the elevated T-maze, a new animal model of anxiety. Brain Res Bull.

[50] Johnson PL, Molosh A, Fitz SD, Arendt D, Deehan GA, Federici LM (2015). Pharmacological depletion of serotonin in the basolateral amygdala complex reduces anxiety and disrupts fear conditioning. Pharm Biochem Behav.

[51] Freitas RL, Uribe-Mariño A, Castiblanco-Urbina MA, Elias-Filho DH, Coimbra NC (2009). GABAA receptor blockade in dorsomedial and ventromedial nuclei of the hypothalamus evokes panic-like elaborated defensive behaviour followed by innate fear-induced antinociception. Brain Res.

[52] Ullah F, dos Anjos-Garcia T, dos Santos IR, Biagioni AF, Coimbra NC (2015). Relevance of dorsomedial hypothalamus, dorsomedial division of the ventromedial hypothalamus and the dorsal periaqueductal gray matter in the organization of freezing or oriented and non-oriented escape emotional behaviors. Behav Brain Res.

[53] Ullah F, dos Anjos-Garcia T, Mendes-Gomes J, Elias-Filho DH, Falconi-Sobrinho LL, Freitas RLde (2017). Connexions between the dorsomedial division of the ventromedial hypothalamus and the dorsal periaqueductal grey matter are critical in the elaboration of hypothalamically mediated panic-like behaviour. Behav Brain Res.

[54] Lammers JHCM, Kruk MR, Meelis W, van der Poel AM (1988). Hypothalamic substrates for brain stimulation-induced patterns of locomotion and escape jumps in the rat. Brain Res.

[55] Phelps EA, LeDoux JE (2005). Contributions of the amygdala to emotion processing: from animal models to human behavior. Neuron.

[56] LeDoux JE (2000). Emotion circuits in the brain. Annu Rev Neurosci.

[57] Chan-Palay V (1976). Serotonin axons in the supra- and subependymal plexuses and in the leptomeninges; their roles in local alterations of cerebrospinal fluid and vasomotor activity. Brain Res.

[58] Hickman D, Fitz S, Bernabe C, Caliman I, Haulcomb M, Federici L (2016). Evaluation of low versus high volume per minute displacement CO2 methods of euthanasia in the induction and duration of panic-associated behavior and physiology. Animals.

[59] Burkholder TH, Niel L, Weed JL, Brinster LR, Bacher JD, Foltz CJ (2010). Comparison of carbon dioxide and argon euthanasia: effects on behavior, heart rate, and respiratory lesions in rats. J Am Assoc Lab Anim Sci.

[60] Oikawa S, Hirakawa H, Kusakabe T, Nakashima Y, Hayashida Y (2005). Autonomic cardiovascular responses to hypercapnia in conscious rats: the roles of the chemo- and baroreceptors. Auton Neurosci.

[61] Forsyth JP, Eifert GH, Canna MA (2000). Evoking analogue subtypes of panic attacks in a nonclinical population using carbon dioxide-enriched air. Behav Res Ther.

[62] Spiacci A, Vilela-Costa HH, Sant’Ana AB, Fernandes GG, Frias AT, da Silva GSF (2018). Panic-like escape response elicited in mice by exposure to CO2, but not hypoxia. Prog Neuro-Psychopharmacol Biol Psychiatry.

[63] Johnson PL, Samuels BC, Fitz SD, Federici LM, Hammes N, Early MC (2012). Orexin 1 receptors are a novel target to modulate panic responses and the panic brain network. Physiol Behav.

[64] Saito YC, Tsujino N, Abe M, Yamazaki M, Sakimura K, Sakurai T (2018). Serotonergic input to orexin neurons plays a role in maintaining wakefulness and REM sleep architecture. Front Neurosci.

[65] Imeri L, de Simoni MG, Giglio R, Clavenna A, Mancia M (1994). Changes in the serotonergic system during the sleep-wake cycle: Simultaneous polygraphic and voltammetric recordings in hypothalamus using a telemetry system. Neuroscience.

[66] Houdouin F, Cespuglio R, Jouvet M (1991). Effects induced by the electrical stimulation of the nucleus raphe dorsalis upon hypothalamic release of 5-hydroxyindole compounds and sleep parameters in the rat. Brain Res.

[67] Eggermann E, Bayer L, Serafin M, Saint-Mleux B, Bernheim L, Machard D (2003). The wake-promoting hypocretin-orexin neurons are in an intrinsic state of membrane depolarization. J Neurosci.

[68] Chowdhury S, Yamanaka A (2016). Optogenetic activation of serotonergic terminals facilitates GABAergic inhibitory input to orexin/hypocretin neurons. Sci Rep.

[69] Stamp JA, Semba K (1995). Extent of colocalization of serotonin and GABA in the neurons of the rat raphe nuclei. Brain Res.

[70] Prouty EW, Chandler DJ, Waterhouse BD (2017). Neurochemical differences between target-specific populations of rat dorsal raphe projection neurons. Brain Res.

[71] Fu W, Le Maître E, Fabre V, Bernard J-F, David Xu Z-Q, Hökfelt T (2010). Chemical neuroanatomy of the dorsal raphe nucleus and adjacent structures of the mouse brain. J Comp Neurol.

[72] Jayaprakash N, Wang Z, Hoeynck B, Krueger N, Kramer A, Balle E (2016). Optogenetic interrogation of functional synapse formation by corticospinal tract axons in the injured spinal cord. J Neurosci.

[73] Tye KM, Prakash R, Kim S-Y, Fenno LE, Grosenick L, Zarabi H (2011). Amygdala circuitry mediating reversible and bidirectional control of anxiety. Nature.

[74] Adhikari A, Lerner TN, Finkelstein J, Pak S, Jennings JH, Davidson TJ (2015). Basomedial amygdala mediates top-down control of anxiety and fear. Nature.

[75] Azim E, Fink AJP, Jessell TM (2014). Internal and external feedback circuits for skilled forelimb movement. Cold Spring Harb Symp Quant Biol.

[76] Kwon J-T, Nakajima R, Kim H-S, Jeong Y, Augustine GJ, Han J-H (2014). Optogenetic activation of presynaptic inputs in lateral amygdala forms associative fear memory. Learn Mem.

[77] Ciocchi S, Passecker J, Malagon-Vina H, Mikus N, Klausberger T (2015). Selective information routing by ventral hippocampal CA1 projection neurons. Science.

[78] Jennings JH, Sparta DR, Stamatakis AM, Ung RL, Pleil KE, Kash TL (2013). Distinct extended amygdala circuits for divergent motivational states. Nature.

[79] Sato TK, Häusser M, Carandini M (2014). Distal connectivity causes summation and division across mouse visual cortex. Nat Neurosci.

[80] Li X, Yamawaki N, Barrett JM, Körding KP, Shepherd GMG (2018). Scaling of optogenetically evoked signaling in a higher-order corticocortical pathway in the anesthetized mouse. Front Syst Neurosci.

[81] Johnson PL, Shekhar A (2006). Panic-prone state induced in rats with GABA dysfunction in the dorsomedial hypothalamus is mediated by NMDA receptors. J Neurosci.

[82] Molosh AI, Johnson PL, Fitz SD, Dimicco JA, Herman JP, Shekhar A (2010). Changes in central sodium and not osmolarity or lactate induce panic-like responses in a model of panic disorder. Neuropsychopharmacology.

[83] Peyron C, Petit J-MM, Rampon C, Jouvet M, Luppi P-HH (1998). Forebrain afferents to the rat dorsal raphe nucleus demonstrated by retrograde and anterograde tracing methods. Neuroscience.

[84] Taylor CB, Sheikh J, Agras WS, Roth WT, Margraf J, Ehlers A (1986). Ambulatory heart rate changes in patients with panic attacks. Am J Psychiatry.

[85] Margraf J, Taylor CB, Ehlers A, Roth WT, Agras WS (1987). Panic attacks in the natural environment. J Nerv Ment Dis.

[86] de Beurs E, Garssen B, Buikhuisen M, Lange A, van Balkom A, Van Dyck R (1994). Continuous monitoring of panic. Acta Psychiatr Scand.

[87] Kessler RC, Wai TC, Jin R, Ruscio AM, Shear K, Walters EE (2006). The epidemiology of panic attacks, panic disorder, and agoraphobia in the National Comorbidity Survey Replication. Arch Gen Psychiatry.

